# Reversible Photoalignment of Azobenzene in the SURMOF HKUST-1

**DOI:** 10.1021/acs.jpclett.1c02489

**Published:** 2021-09-09

**Authors:** Tillmann Koehler, Ina Strauss, Alexander Mundstock, Jürgen Caro, Frank Marlow

**Affiliations:** † Max-Planck-Institut für Kohlenforschung, 45470 Mülheim a. d. Ruhr, Germany; ‡ Leibniz Universität Hannover, Welfengarten 1, 30167 Hannover, Germany; § Center for Nanointegration Duisburg-Essen (CENIDE), University of Duisburg-Essen, 47057 Duisburg, Germany

## Abstract

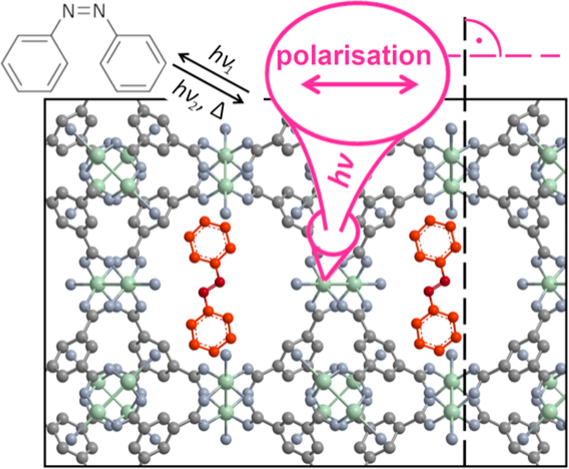

Azobenzene guest molecules in the metal–organic framework structure HKUST-1 show reversible photochemical switching and, in addition, alignment phenomena. Since the host system is isotropic, the orientation of the guest molecules is induced via photo processes by polarized light. The optical properties of the thin films, analyzed by interferometry and UV/vis spectroscopy, reveal the potential of this alignment phenomenon for stable information storage.

One of the most successful applications of the photochromic molecule azobenzene (AB) and its derivatives is its use as a command layer for liquid crystal displays (LCDs).^1−3^ Here, the molecules are located in a polymer environment and linearly polarized light is used to generate an alignment of the molecule’s long axis. This orientation is then transferred to the liquid crystalline layer being forced into a special, e.g., twisted, arrangement in the LCD by the command layers. The photoalignment is rooted in the repeated isomerization of the molecules between the trans (t-AB) and cis (c-AB) states under irradiation which brings the molecular ensemble in a photodynamical equilibrium state.^4,5^ All molecules are continuously switched back and forth until they are, by chance, oriented with their long axis perpendicular to the polarization direction of the light (Figure 1).^6^

**Figure 1:**
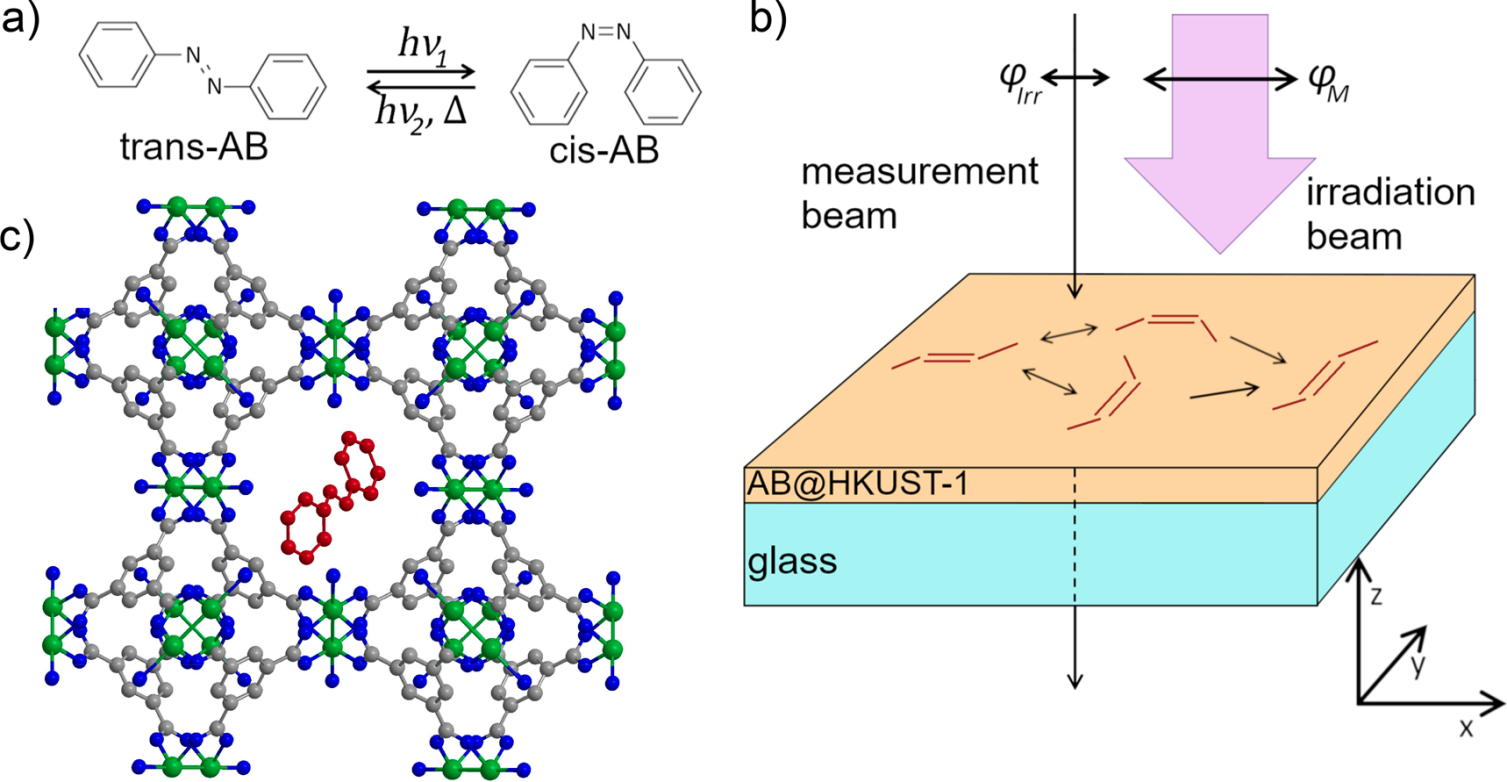
a) Isomerization of AB. b) Experimental scheme and mechanism of photoalignment: The irradiation beam switches the AB; another beam is used to measure absorption. The reorientation is shown on a simplified AB model with the NN double bond and adjacent bonds: AB continuously isomerizes between trans and cis. Excitation of t-AB oriented perpendicular to irradiation polarization is unlikely, and therefore, after multiple cycles this orientation is preferentially observed. c) An AB molecule (red) in the HKUST-1 structure with Cu (green), O (blue), and C (gray) atoms.

The effect of AB photoisomerization (Figure 1a) has been investigated intensely in solution^7^ and porous environments, e.g., in molecular sieves.^8^ In the latter case, the orientation of the transition moment parallel to the t-AB long axis, combined with the constrained anisotropic environment, can be exploited to generate systems with reversibly switchable optical parameters. A large photoswitchable birefringence in AB@zeolite systems has been reported.^9^ However, alignment of AB molecules can also be achieved by irradiation with linearly polarized light in isotropic environments (Figure 1b).^10^

One drawback of the molecular sieves as porous host for optical applications, despite their promising properties in many regards, is the inability to produce macroscopic crystals or homogeneous thin films. Experiments on zeolite crystals therefore suffer from scattering and low reproducibility of the synthesis. One way to circumvent this limitation is by replacing the host with surface mounted metal–organic frameworks (SURMOFs). For this material class, the ability to produce homogeneous thin films on a substrate has been demonstrated on many different MOF types,^11^ e.g., using layer-by-layer synthesis procedures.^12^ Recent reviews of AB@MOF systems are available.^13,14^ In pioneering papers, the reversible AB photoisomerization has been exploited as a molecular valve to close and open the pores of an AB@SURMOF membrane to switch gas transport.^15−17^ Furthermore, AB attached as a side group to the linker can be selectively switched using circularly polarized light.^18^ An especially good photoisomerization behavior of AB was shown in the 3D pores of an HKUST-1 type thin film.^19^ This is possible since two of the framework’s three cavity types (with diameters of 1.4 nm, 1.1 and 0.5 nm^20^) are not only spacious enough to accommodate AB but also provide a free volume bigger than 0.12 nm^3^, which is needed for photoisomerization of AB.^21^

In the present Letter, we describe a geometrically well-defined AB@HKUST-1 composite system (Figure 1c) with the focus of generating anisotropic optical properties via photoalignment. Since the host crystal is isotropic, we induce an orientation of the guest molecules only via photoalignment, similar to the applications of AB as a command layer for LCDs. Regarding potential applications, the photoalignment has a big advantage over different photoisomerized states that it does not suffer under the inherent metastability of one of the states and offers easy coupling to birefringence. Furthermore, in this paper, the optical properties of the thin films will be analyzed with angular dependent interferometry (ADI)^22^ with high accuracy and the stability times of the cis isomer will be determined.

The HKUST-1 MOF-films were prepared layer-by-layer from benzene-1,3,5-tricarboxylic acid and Cu(II) acetate precursor on FTO glass substrate by a spraying technology,^23,24^ resulting in SURMOFs of optical quality. For AB guest loading, the samples were immersed in an ethanolic solution of AB for 72 h at 60 °C. X-ray diffraction showed good agreement with the literature^19,25^ and our previous work on this system (Figure S1).^26^ The nominal loading of the samples was about 1.5 molecules per unit cell, as concluded from UV/vis spectra (see Supporting Information).

The thin films appear slightly colored and expose distinct interference fringes (Fabry–Perot oscillations) in the UV/vis spectra that can be used to determine the film thickness *d* and refractive index *n*. In ADI^22^ this is realized by turning the sample in the spectrometer (for details see the Supporting Information). The interference extrema are blue-shifted with increasing incidence angle α (Figure 2). A multistep process was employed for the data evaluation, as in ref (22). The thickness of our thin films turned out to be *d* = 297 nm ± 12 nm with a refractive index *n* = 1.43. Moreover, the clear occurrence of the Fabry–Perot oscillations shows the excellent SURMOF film quality.

**Figure 2:**
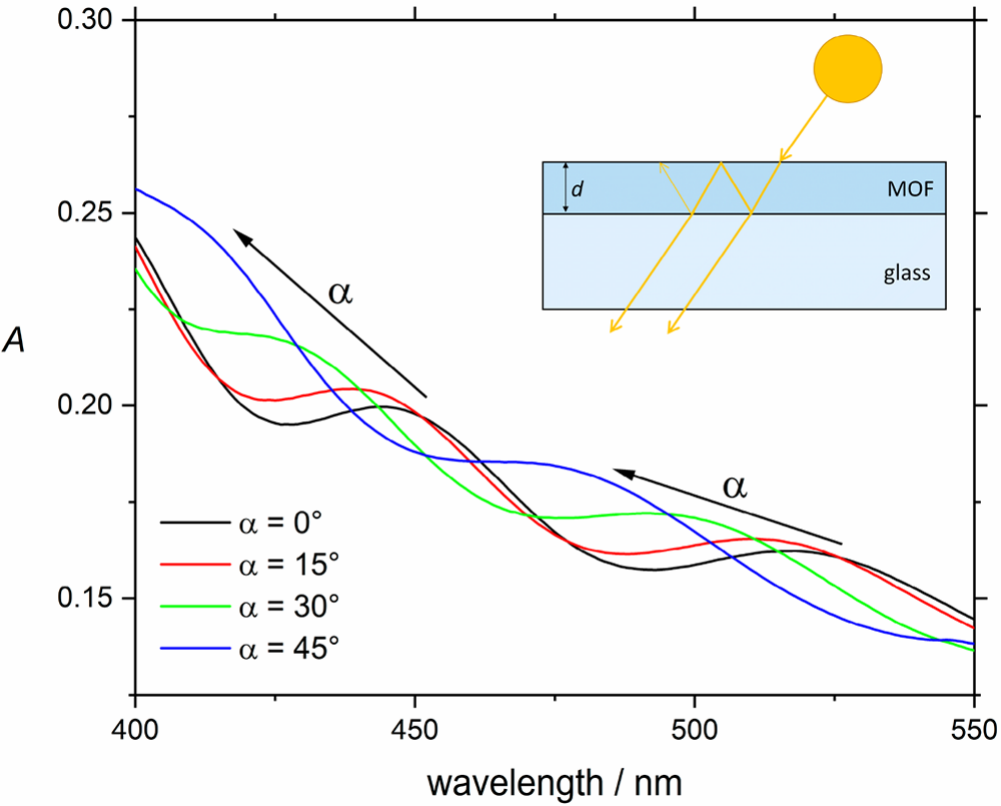
Fabry–Perot oscillations in the absorbance spectrum of the AB@HKUST-1 film. The extrema are blue-shifted with increasing incidence angle α and can be used to determine the optical parameters of the film.

The photoisomerization of the AB guest was investigated with UV/vis spectroscopy. The sample was irradiated alternately with visible light at 443 nm and UV light at 365 nm. The most concise way to visualize the isomerization reaction is to plot the change in absorbance Δ*A* with reference to a measurement on the same sample (Figure 3). Here, the state after the first irradiation with visible light was chosen as reference. A nearly reversible change between two distinct types of spectra, depending on the last irradiation wavelength, was observed.

**Figure 3:**
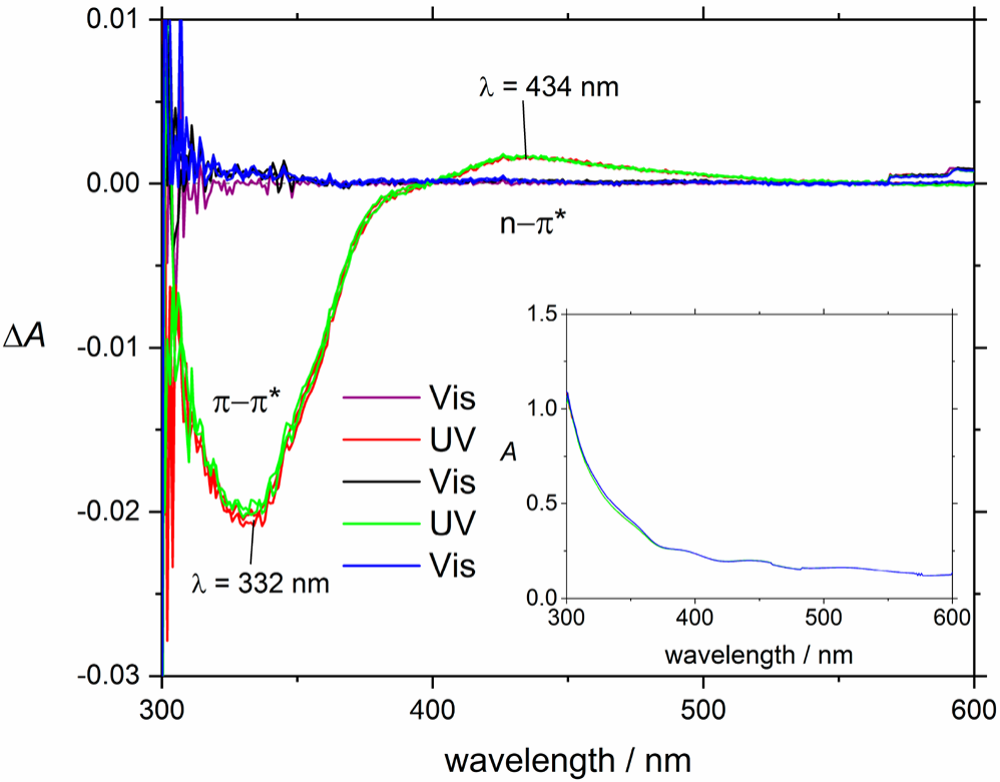
Absorbance change after repeated irradiation with visible and UV light for 3 min. Two spectra were measured after each irradiation, but they varied only little. The dominant π–π* and weaker n-π* bands of the AB are observed at their expected positions. Absolute absorbance spectra are shown in the inset.

As known from the literature,^4,5^ the changes can be attributed to an isomerization of the AB guest molecule by the position of the peaks. The biggest change around 332 nm is negative and corresponds to a loss of intensity of the π–π* band of the t-AB. The π–π* band of the c-AB is expected at much lower wavelengths (and with lower intensities) and is not within the measurement range. The other peak at 434 nm corresponds to the n−π*-band, where the t-AB and c-AB positions are found at roughly the same wavelength. The n−π* intensity of the c-AB, however, is larger than that of the t-AB by a factor of about three and therefore a positive change after irradiation with UV light is expected and was also observed. All positions and intensities depend on the solvent and environment of the AB molecule and small variations from the literature values can likely be attributed to such effects.

One important parameter in evaluating a photoswitchable system is its stability. Typically, the metastable c-AB decays to t-AB in a wide range of time scales, depending heavily on the local environment. We have investigated this process by first irradiating the sample with UV light and then taking UV/vis measurements in regular intervals Δ*t*. The resulting spectra were evaluated at the minimum of the Δ*A* spectrum in the π–π* band with results shown in Figure 4. In all cases, the trend can be fitted well with an exponential function, but the fit is significantly improved if the first data point is neglected. The decay constant *t*_1_ of the exponential function shows a linear dependence on measurement frequency Δ*t*^–1^, similar as in ref (9). This behavior is caused by the influence of the measurement light. Extrapolating this trend toward zero removes the influence of the measurement and yields *t*_1_ = 260 min ± 34 min. This result means c-AB relaxes faster in HKUST-1 than it does in solution, where it has a half-life of around 2 days.^5,27^ AB in molecular sieves shows comparable or a little longer stability times than the values we report here.^9^

**Figure 4:**
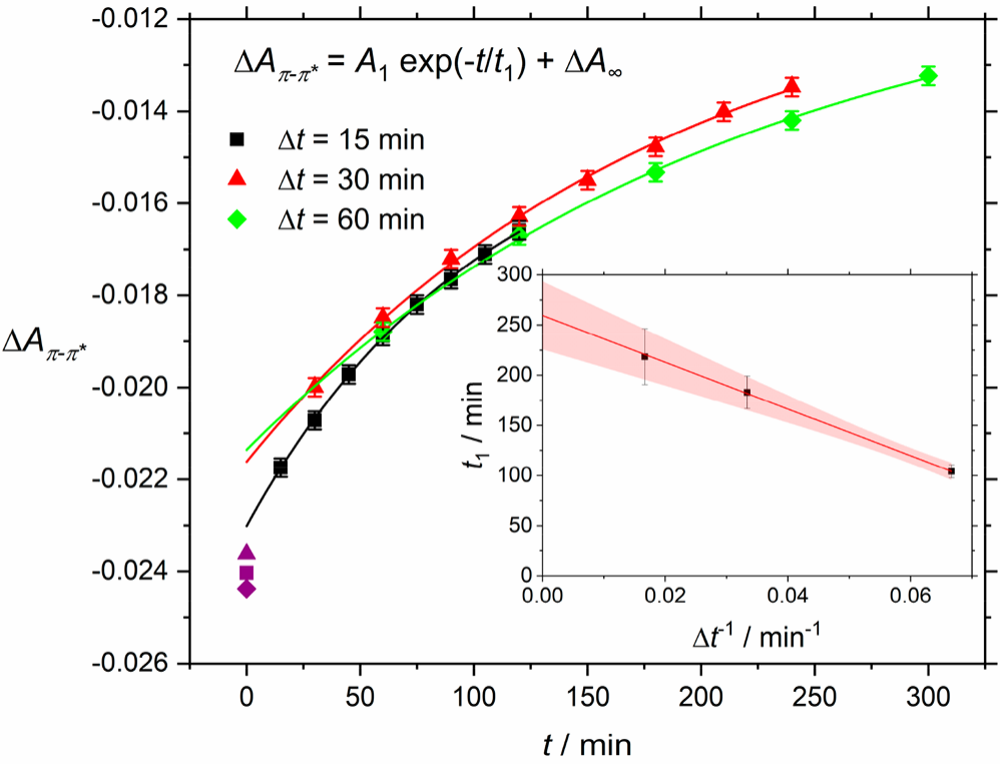
Stability analysis of the cis state. Change in absorbance Δ*A* in the π–π* band plotted against time after irradiation with different Δ*t* between the measurement points and exponential fits. Error bars were estimated from the noise of the absorbance data. Inset: Dependence of the decay constants *t*_1_ vs measurement frequency Δ*t*^–1^. A linear fit together with the 95% confidence area is shown.

During the data evaluation, we excluded the first data points for an improved exponential fit in Figure 4, meaning that the relaxation in the first time period follows a slightly different behavior than during the rest of the measurement. Such effects have been reported before as result of sterically hindered c-AB, e.g., in a polymer environment.^28^ We believe that a fraction of the AB molecules cannot relax completely, because of the interaction with the HKUST-1 matrix.

Following the motivation given above, the switching experiments were finally modified to be sensitive to the orientation of the AB molecules. To our knowledge, this question has never been investigated in an isotropic porous guest–host system. The sensitivity can be achieved by inserting a linear polarization filter in front of the sample (orientation φ_*M*_) and the UV lamp (orientation φ_*Irr*_), respectively. Figure 5 shows measurements on AB@HKUST-1 where after each irradiation two absorption measurements were performed: one polarized horizontally and one vertically. After irradiation with nonpolarized light, the two polarizations show almost no difference, as expected, since the AB is distributed isotropically. After irradiation with linearly polarized UV light, however, higher absorption in the direction perpendicular to the polarization direction of the irradiation light was observed. This effect is consistent with an enhanced orientation of AB molecules in the perpendicular direction by photoalignment. For additional measurements, see section 2.5 of the Supporting Information.

**Figure 5:**
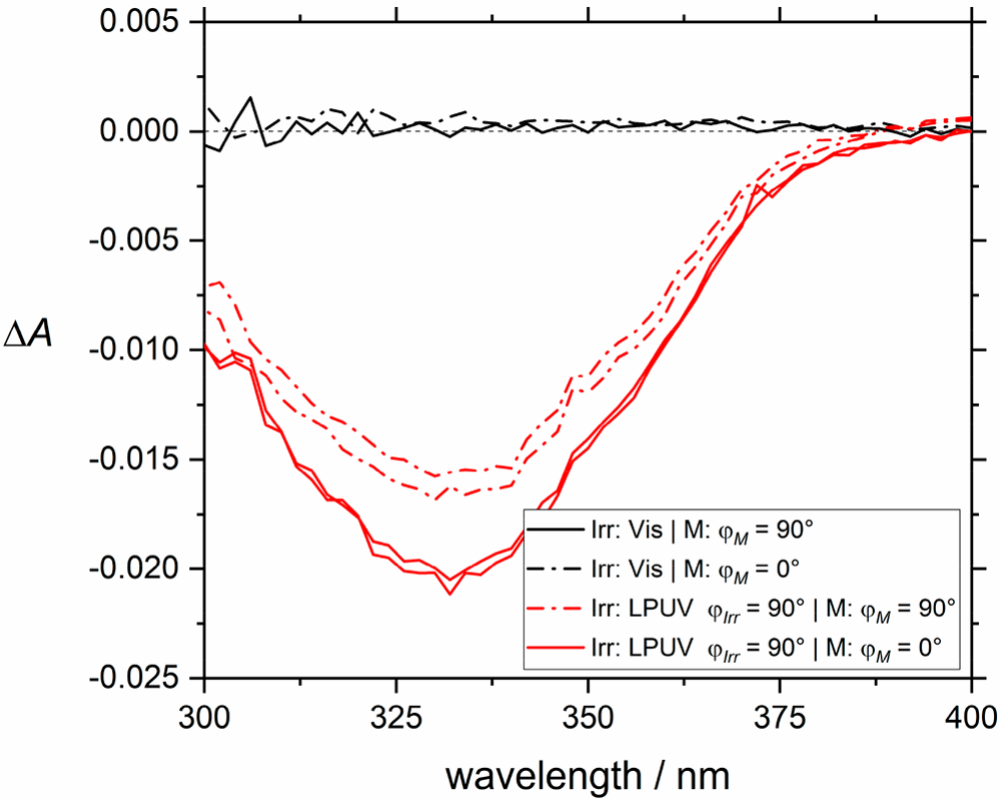
Photoalignment in AB@HKUST-1: absorbance change of the π–π* band of light polarized vertically (continuous lines) and horizontally (dotted lines). After irradiation with nonpolarized light, no difference between polarizations was observed, while after irradiation with linearly polarized UV (LPUV) light a clear difference can be seen.

The influence of the measurement on the aligned state needs to be taken into consideration just as carefully as the influence of the measurement light on the isomer concentrations. In Figure 6 this influence is monitored by two measurements taken in each polarization direction in alternating order. Only a small difference between these two was found. The measurement range and hereby duration of exposure was nevertheless further reduced to a single spectral data point in the following measurements. Additional angles between the perpendicular and parallel polarization were measured to be able to check the data consistency. The results shown in Figure 6 can be fitted in all cases with a cos^2^ function, thus, confirming photoalignment of the AB guest molecules.^29^

**Figure 6:**
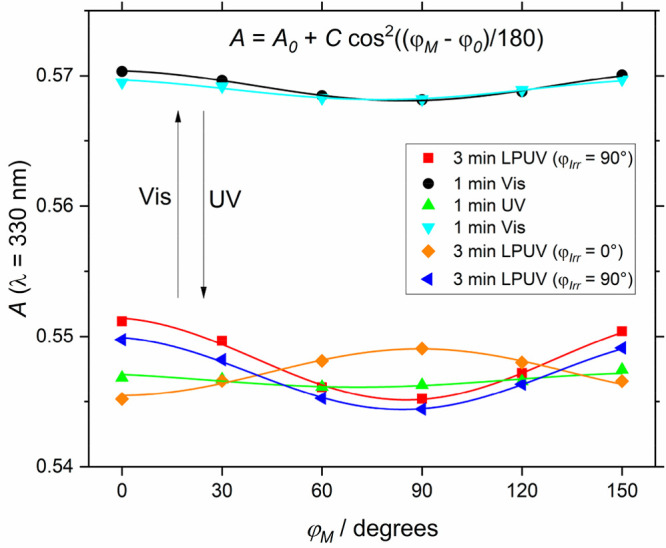
Polarized absorbance at λ = 330 nm after 1 min of irradiation with the light type indicated in the plot legend. After each irradiation, six data points at different polarization directions φ_*M*_ were collected and fitted with a cos^2^ function. The highest absorbance is observed perpendicular to the linearly polarized light φ_*Irr*_, which is consistent with AB aligned in this direction.

The sequence of measurements is shown by the order in the plot legend in Figure 6, from top to bottom. Considering this, the results reveal two additional surprising characteristics. First, some degree of orientation is preserved even after irradiation with nonpolarized light, both visible and UV. However, every additional irradiation does reduce the degree of orientation. This is seen by the difference of the cyan and the black curve, between which an additional switching step with nonpolarized UV and visible light was performed. Furthermore, an intermediate step of irradiation with visible light was not always needed for the alignment. The state of preferred alignment of the AB molecule in the vertical direction (orange) was turned around directly toward a preferential orientation in the horizontal (blue).

The strength of the alignment effects can be estimated by comparison with the total photochemical changes. In the data fit formula (see Figure 6), the change in absorption due to the isomerization is described by the difference in *A*_0_ between the measurements after irradiation with UV to the one with visible light, while the amplitude *C* of the cosine is a measure of the photoalignment. *A*_0_ is about four times larger than *C*, suggesting that effectively 1/4 of the AB molecules are photochemically reoriented inside the pores. This proportion can likely be enhanced by further optimization of the irradiation conditions.

As already noted, photoalignment of AB has been observed before, mostly for AB derivatives anchored on one end, e.g., to a polymer.^29^ No alignment is expected for AB in solution, as an oriented state would quickly be lost due to thermal redistribution. The occurrence of photoalignment inside MOF pores, which are sometimes deemed to behave like a “solid solvent”,^30^ is therefore by no means trivial. We believe that the guest–host interactions are an important factor in stabilizing the system. This may bypass a general drawback of many photoalignable systems with separated small molecules where the aligned states are not stable. In our system, we have not observed any relaxation process of the alignment degree up to now.

Stability of the cis state for about 4 h was found. This was plenty enough to perform photoisomerization experiments, but these stability times are too low for applications in data storage. The photoaligned states are, however, not influenced by the same processes. This means information can be much better stored in the orientation of the t-AB, which is not lost after thermal isomerization. The relaxation of the photoalignment toward an isotropic distribution is described by Brownian motion and the interaction with the environment.^29^

The found refractive index of *n* = 1.43 for the SURMOF film requires a closer look. Redel et al. reported the refractive index of HKUST-1 thin films increases from *n* = 1.39 to *n* = 1.55–1.6 by the presence of water inside the pores.^31^ Since our refractive index for the composite AB@HKUST-1 is only marginally bigger than in the fully activated state, it is once again indicated that only small amounts of guest molecules are present. This and the small thickness are responsible for the small optical changes observed in this work. These changes are however in the same range as similar systems shown in the literature.^14,19^ Differences can likely be attributed to film thickness, loading procedures, and wavelength of light used for the photoisomerization. The optical effects can certainly be increased with further optimization of these parameters. Some inherent limitations might be foreseen in the loading capacity and in film thickness because of scattering. From the molecular view, loading up to 32 molecules/u.c. seem to be possible if one assumes a dominant adsorption interaction of AB and the BTC. Optical scattering of the MOF films was not visible up to now.

In addition, we believe that additional strategies for increasing the optical switching effects are needed and photoalignment can be one of them. It can, for example, be followed up by birefringence read-out, where small changes in AB@host systems have already been used before to induce large transmission changes at specific wavelengths.^8,9^ This would also solve the inherent problem of measurement influence, because read-out wavelengths can be chosen outside of the typical absorption range of AB. We will continue this work by conducting similar experiments on AB@MOF systems.

In this work, the geometry of the switching and measurement light was kept as simple as possible. Both hit the sample parallel to the surface normal and only the polarization direction was varied. It is, however, also possible to irradiate and measure the sample from different angles. In such a case the orientation of the AB molecules needs to be described by x, y, and z components. Additional experiments have already shown that the alignment is not limited to the MOF’s film plane. It is even possible to align the AB with nonpolarized light in the direction of the light beam, as was described before in AB@Polymer systems.^6^ 3D anisotropy, instead of the often discussed anisotropy inside a plane, could pave the way for many interesting applications, such as data storage, multicommand switching geometries and neuromorphics.

In summary, photoalignment of AB in the pores of the HKUST-1 SURMOF was shown to be possible, efficient, and stable. The molecules are oriented perpendicular to the polarization direction of switching light. To our knowledge, this is the first time that this has been observed for noncovalently attached azobenzene. Reversible switching between two such aligned states works and more sophisticated switching geometries are possible. Because of the large variety of MOF pore sizes available in the literature, many photoisomerizable molecules can possibly be aligned in a similar way. Moreover, especially enhancement with birefringence switching could lead to a large contrast in the measurable optical effects. Birefringence effects are inherently expected for the photoaligned systems, whereas they are restricted to very special cases when only different isomerization states are used such as in ref (8).

## Supporting Information Available

Experimental details, the XRD measurements, ADI, guest loading calculation, and more details about the cis-lifetime and photoalignment measurements (PDF)
