# Supplementary material for: Reversible Photoalignment of Azobenzene in the SURMOF HKUST-1
Source: J Phys Chem Lett. 2021 Sep 9;12(36):8903–8. doi: 10.1021/acs.jpclett.1c02489 (PMC8450931; doi:10.1021/acs.jpclett.1c02489)
Supplement: Supplementary file 1 — jz1c02489_si_001.pdf [file jz1c02489_si_001.pdf]

Supporting Information

Reversible Photoalignment of Azobenzene in the SURMOF HKUST-1

Tillmann Koehler,[a] Ina Strauss [b] , Alexander Mundstock, [b] Jürgen Caro [b] and Frank Marlow* [a,c]

[a] Max-Planck-Institut für Kohlenforschung, Kaiser-Wilhelm Platz 1, 45470 Mülheim a. d. Ruhr, Germany.

[b] Leibniz Universität Hannover, Welfengarten 1, 30167 Hannover, Germany

[c] Center for Nanointegration Duisburg-Essen (CENIDE), University of Duisburg-Essen, 47057 Duisburg, Germany

* Corresponding author. Email: marlow@mpi-muelheim.mpg.de

Inhalt

1 Experimental details 2

1.1 Fabrication of the AB@MOF films 2

1.2 General sample characterization 2

1.3 Photoisomerization 2

1.4 UV/VIS spectroscopy 3

2 Additional Results 3

2.1 X-ray diffraction 3

2.2 Analysis of the films with angular dependent interferometry 3

2.3 Guest loading calculation 5

2.4 Decay constant investigations 6

2.5 Photoalignment 7

3 References 9

1 Experimental details

1.1 Fabrication of the AB@MOF films

The HKUST-1 MOF-films (Cu_3BTC_2) were fabricated layer-by-layer on 1.9 cm x 1.9 cm soda-lime glass pieces. The glass was coated with a fluorine doped tin oxide (FTO) layer and was purchased from Sigma-Aldrich (TEC7).

After an initial cleaning step (ultrasonification in ethanol (ROTIPURAN, ≥ 99.8 %, p.a., Roth) for 10 min), one substrate at a time was vertically fixed on a motorized mount with a distance of 12 cm to the spray nozzle (airbrush gun from Conrad Electronic) and rotated throughout the whole process. HKUST-1 layer preparation was done at RT in cycles comprised of four steps each:

1) 20 seconds of linker deposition (0.2 mM ethanolic solution of benzene-1,3,5-tricarboxylic acid (BTC, 95 %, Sigma-Aldrich))
2) 20 seconds of washing (ethanol)
3) 20 seconds of metal deposition (1 mM of copper(II) acetate monohydrate (ACS, 98+ %, Alfa Aesar) in ethanol)
4) 20 seconds of washing (ethanol).

This procedure was repeated until the MOF film's thickness reached the aspired value (e.g. around 3 h for 300 nm). Finally, the resulting specimens were dried at RT overnight. For guest loading, the samples were stored in an azobenzene containing ethanol solution (0.01 M) for 72 h at 60 °C and subsequently dipped into fresh ethanol in order to wash away all the excess AB. After this procedure, the backside of the glasses was wiped with an ethanol-soaked tissue to remove any excess azobenzene. Some remaining solvent molecules in the pores cannot be excluded, but we have found no indication of it during our investigations.

1.2 General sample characterization

The X-ray powder pattern for qualitative phase analysis was collected on a Rigaku SmartLab equipped with a rotating anode (9 kW, 45 kV, 200 mA) in Bragg-Brentano-geometry (Cu $K_{\alpha1,2}$: 1.54 Å). An elliptical multilayer mirror was used for generating high-resolution radiation. Data were collected with a HyPix-3000 multi-dimensional detector in 1D mode. Data were collected in the range between 3° and 25° in 2θ with a step width of 0.01°. 8 scans were collected and summed after data collection.

1.3 Photoisomerization

The cis- and trans-azobenzene dominated photostationary states were switched with a *Chameleon Series CU6* (Nitecore) UV/VIS source. The utilized settings were UV-light at 365 nm (3 W) and visible (white) light (4 W) at maximum intensity, with a dominant line at 443 nm; referred to as "UV" and "Vis". The UV/VIS source was placed on a holder in the spectrometer sample chamber in a distance of 5 cm from the sample. In this position, about 70 % of the intensity is directed at the sample ($(1.8\ cm)^2$) and most of the 3 W x 10 % (efficiency) reaches the sample. This corresponds to about 4 J/cm^2 after 1 min and 12 J/cm^2 after 3 min of irradiation. Linearly polarized light (LPUV) of the same source was obtained by fixing a polarizing filter in the light beam.

1.4 UV/VIS spectroscopy

UV/VIS Measurements were performed on a *Cary 5G* spectrometer (Agilent) in double beam mode. Both, the sample and the polarization filters were placed inside the sample chamber. The sample glass with the thin film was placed standing upright, with only minimal contact at the bottom, and could be turned by angles $\alpha = 15°$. During the photoisomerization and photoalignment measurements, both, the measurement and irradiation beam, hit the sample perpendicular to its surface. For photoalignment investigations, the linear polarizing filter was placed in front of the sample and could be rotated by steps of $\varphi_M = 15°$.

A measurement of the empty sample chamber was always recorded at the start of the measurement procedure and used as a baseline. Two scans in the chosen wavelength range are usually performed after each irradiation to control for the influence of the measurement and each takes about 40 s. A wait time of one minute after each irradiation was imposed, to avoid the influence of faster processes.

2 Additional Results

2.1 X-ray diffraction

The diffraction data of the AB@HKUST-1 thin film are shown in Figure S1. A comparison with HKUST-1 powder data[1] shows strong coincidence. The diffractogram also corresponds well with previous investigations on AB@HKUST-1 SURMOFs.[2, 3] The most intense peaks are observed in the 222- and 111-direction.

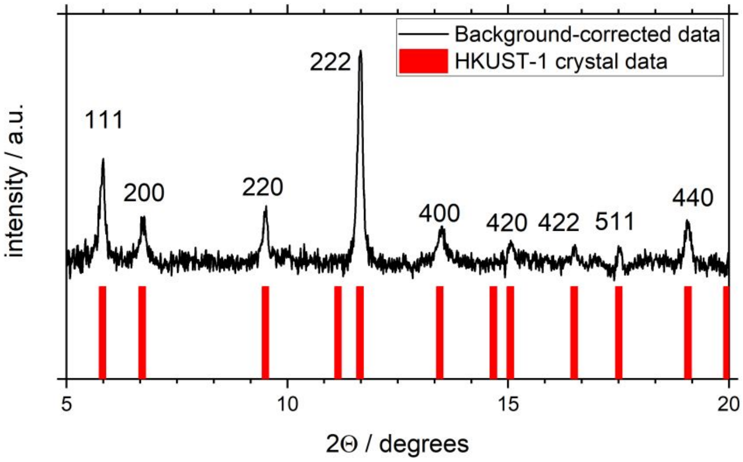

Figure S1. The XRD-data of AB@HKUST-1 (black) and a comparison with ref. [1] (red).

2.2 Analysis of the films with angular dependent interferometry

ADI allows the simultaneous determination of thickness *d* and refractive index *n* of the films. The four-step procedure follows a method described by Konjhodzic et al.[4] and is shown in Figure S2.

First, the measured absorbance for $\alpha = 0° - 45°$ was fitted with a double exponential function. This was done because analysis of the residuals for its peak positions is easier and much more accurate than doing so in the initial. The resulting peak positions are shown in Figure S2b. Their wavenumber is linearly related to their order as shown in Figure S2c. The attribution of the order was done by trial and error with the

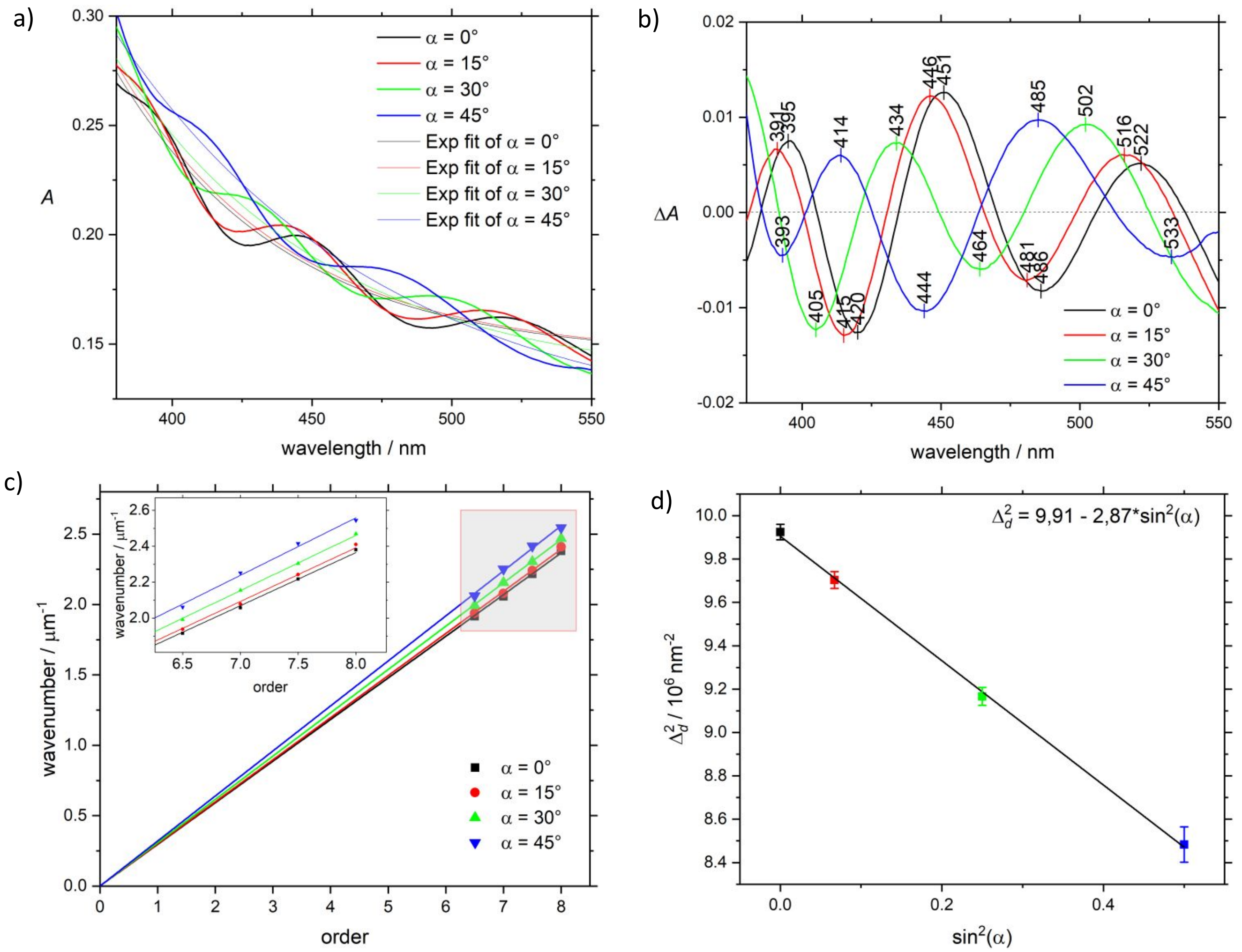

Figure S2. Analysis of an AB@HKUST-1 thin film for its thickness d and refractive index n. a) Initial UV/VIS measurements and exponential fit, b) residual of the fit and deduced peak positions, c) linear fit of the wavenumber of these peak positions to the order, and d) linear fit of the optical path difference Δ*d* to $\sin^2(\alpha)$ according to eq. S2.

condition that the wavenumber must be zero at zero order. The squared, inverse slope of each linear fit in Figure S2c was then plotted against $\sin^2$ of the incidence angle α. The inverse slope gives the thickness-dependent part of the optical path difference Δ_d, which is connected to α by Equation S1. A linear fit of the data points was possible this way.

$$\Delta_d = 2d\sqrt{n^2 - \sin^2 \alpha} \qquad \text{(S1)}$$

The thin film parameters and their errors can finally be calculated from the linear fit parameters Y_0 (Y-axis intercept) and *m* (slope) indicated in Figure S2d:

$$d = \frac{\sqrt{m}}{2} = \frac{\sqrt{2.87 \cdot 10^6 \text{ nm}^{-2}}}{2} = 846 \text{ nm}$$

$$\Delta d = \frac{\Delta m}{4\sqrt{m}} = \frac{6.04 \cdot 10^4 \text{ nm}^{-2}}{4\sqrt{m}} = 8.91 \text{ nm}$$

$$n = \sqrt{\frac{Y_0}{m}} = \sqrt{\frac{9.91 \cdot 10^6 \text{ nm}^{-2}}{2.87 \cdot 10^6 \text{ nm}^{-2}}} = 1.86$$

$$\Delta n = \sqrt{\frac{\Delta Y_0{}^2}{4Y_0 m} + \frac{\Delta m^2 Y_0}{4m^3}} = \sqrt{\frac{\left(1.70 \cdot 10^4 \text{nm}^{-2}\right)^2}{4Y_0 m} + \frac{\left(6.04 \cdot 10^4 \text{ nm}^{-2}\right)^2 Y_0}{4m^3}} = 0.0196$$

The FTO-layer of the substrate under the MOF film needs to be accounted for. The FTO was also analyzed with ADI. The determined thickness d_{FTO} = 550 nm ± 7 nm corresponds exactly to the values given by the manufacturer, while the refractive index n_{FTO} = 2.09 ± 0.02 lays well within the range of expected literature values for FTO[5, 6]. We furthermore assume that the smallest possible oscillation in Figure S2 corresponds to the interference through an effective film consisting of both, the MOF and the FTO film. To isolate only the contribution of the HKUST-1 we employ this 2-film model and calculate n_{MOF} and d_{MOF} in the following way:

$$\boldsymbol{d_{MOF}} = d_{MOF\,+\,FTO} - d_{FTO} = 846 \text{ nm} - 550 \text{ nm} = \mathbf{297\ nm}$$

$$\boldsymbol{n_{MOF}} = \frac{d_{MOF\,+\,FTO}\, n_{MOF\,+\,FTO} - d_{FTO}\, n_{FTO}}{d_{MOF}} = \frac{846 \text{ nm} \cdot 1.86 - 550 \text{ nm} \cdot 2.09}{297 \text{ nm}} = \mathbf{1.43}$$

Error propagation laws result in errors of 12 nm for the thickness.

2.3 Guest loading calculation

The mass loading is estimated from the observed change of absorbance Δ*A* in the π-π*-band (λ = 330 nm) with Lambert-Beer law

$$A(\lambda) = c_{AB} \cdot d \cdot \left(x_{cis} \cdot \varepsilon_{cis}(\lambda) + (1 - x_{cis}) \cdot \varepsilon_{trans}(\lambda)\right).$$

The change in absorbance due to the irradiation results entirely from the change in cis-content x_{cis} (with a value between 0 and 1)

$$\begin{aligned} \rightarrow \Delta A(\lambda) &= c_{AB} \cdot d \cdot \left(\Delta x_{cis} \cdot \varepsilon_{cis}(\lambda) - \Delta x_{cis} \cdot \varepsilon_{trans}(\lambda)\right) \\ &= c_{AB} \cdot d \cdot \Delta x_{cis} \cdot \left(\varepsilon_{cis}(\lambda) - \varepsilon_{trans}(\lambda)\right). \end{aligned} \qquad \text{(S2)}$$

x_{cis} is assumed to be 0 initially and 65 % after irradiation with UV light, according to the results of Müller et al.[2], resulting in an overall change $\Delta x_{cis} = 0.65$. The difference in extinction constant *ε* of both isomers is a material parameter and taken from the literature[7, 8]:

$$\begin{aligned} \varepsilon_{cis}\,(\lambda = 330 \text{ nm}) - \varepsilon_{trans}\,(\lambda = 330 \text{ nm}) &= 2500 \text{ Lmol}^{-1}\text{cm}^{-1} - 22000 \text{ Lmol}^{-1}\text{cm}^{-1} \\ &= -19500 \text{ Lmol}^{-1}\text{cm}^{-1} \end{aligned}$$

The change in absorbance of a sample stored in the dark for more than 2 weeks is shown in Figure S3. This is done because the c-AB will slowly decay back to t-AB (see chapter 2.4) and therefore the initial c-AB content can be assumed to be basically 0. We display the absorbance in reference to an earlier

measurement, in this case to the measurement before any irradiation, and we can read out a maximum change of absorbance in the π-π*-band of

$$\Delta A(\pi\text{ - }\pi^*) = -0.0475.$$

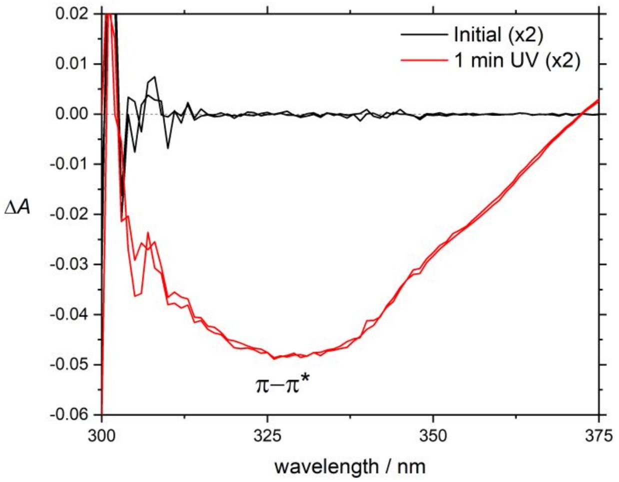

Figure S3. Change in absorbance of an AB@HKUST-1 to the initial state (black) after irradiation with UV light (red). Due to long storage in the dark, the initial c-AB content can be assumed to be close to 0. Two measurements are taken in both cases to control for the measurement influence and show little to no difference.

We can then calculate the AB concentration in the pores, using the previously calculated thickness of the MOF *d* and eq. S2

$$c_{AB} = \frac{\Delta A}{\Delta\varepsilon_{lit}(\pi\text{ - }\pi^*)\cdot d\cdot \Delta x_{cis}} = \frac{-0.0475}{-19500\ \mathrm{Lmol}^{-1}\mathrm{cm}^{-1}\cdot 300\ \mathrm{nm}\cdot 0.65} = 0.125\ \mathrm{molL}^{-1}.$$

Finally, we convert the value into molecules per unit cell of the HKUST-1 with a lattice parameter a = 26.3 Å[9], resulting in

$$\boldsymbol{c} = 0.125\ ^{\mathrm{mol}}/_{\mathrm{L}} \equiv 125^{\mathrm{mol}}/_{\mathrm{m}^3}\cdot N_A\cdot(26.3\text{Å})^3\frac{\text{molecules}}{\text{u.c.}} = \mathbf{1.37}\frac{\text{molecules}}{\text{u.c.}}.$$

This calculation contains estimates for some and measurement uncertainties for all variables and should be taken as an estimate, hence our rounding in the main work to about 1.5 molecules per unit cell.

2.4 Decay constant investigations

For the cis-state stability investigations described in the main text, the system was first switched twice between trans and cis state (Figure S4). Then, the sample was irradiated for 1 min with UV light and the absorbance was measured in regular intervals Δ*t,* as described in the main work. All of these spectra in reference to the same state are shown in red and their absorbance in the π-π*-band increases continuously. The exact absorbance values were determined via the peak analysis function of *Origin 2019*. The data point corresponding to $t = 0$ was neglected here, which improved the fit quality, e.g. from R^2 = 98.7 % to R^2 = 99.9 % for Δt = 30 min. All further analysis steps are discussed in the main work.

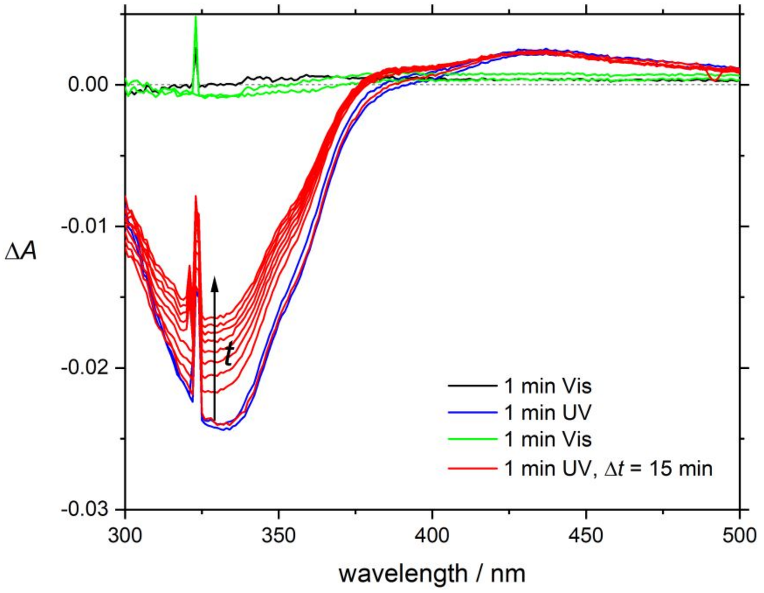

Figure S4. Absorbance change of AB@HKUST-1 used for the decay constant investigation for Δt = 15 min shown in Figure 4 of the main work. The intensity in the π-π*-band increases on the absolute *A*-scale, showing the continuous re-appearance of the t-AB.

2.5 Photoalignment

In this section, additional measurements in the series of Figure 5 will be shown and discussed to further substantiate photoalignment and exclude any measurement influence as cause of the observed effects. In the second part the corresponding fit parameters to the curves in Figure 6 are given.

a) For the first set of photoalignment investigations the polarization directions of measurement and irradiation light were either parallel or perpendicular. In the main work, photoalignment is shown by a few selected curves for clarity sake. More curves of the same measurement series are shown in Figure S5. Displayed in blue are measurements after irradiation with non-polarized UV light. The system was, therefore, switched to the cis-dominated state. Only little differences between the two measurements with different polarizations can be seen in this case. The system was isotropic, just like after irradiation with non-polarized visible light.

The state after irradiation with LPUV light, turned by 90° compared to the main work, is shown in green. In this case, the dash-dotted and continuous lines are exchanged, as the perpendicular molecular alignment arrangement now corresponds to φ_M = 90° and not anymore to φ_M = 0° as for the red curve. This shows another time that our observed effects are indeed due to photoalignment and not due to measurement errors of some kind.

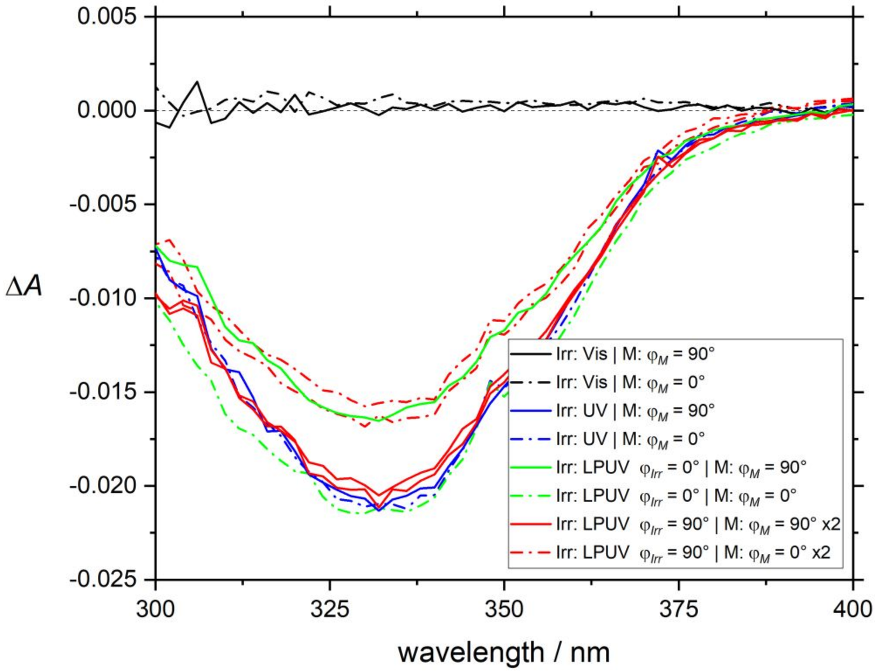

Figure S5. Photoalignment in AB@HKUST-1. All spectra shown as absorbance difference to a single reference state, obtained after irradiation with non-polarized Vis light. Dash-dotted lines refer to measurements taken at φ_M = 0°, while continuous lines refer to those taken with light polarized at φ_M = 90°. In addition to the measurements shown in the main work (Figure 5: black and red), measurements after irradiation with non-polarized UV and LPUV at φ_{Irr} = 90° are shown. All irradiations steps were done by irradiation for 1 min, the measurement sequence is shown in the plot legend.

b) To minimize possible measurement influences on the photoalignment, the observation of the system was reduced to the maximum of the π-π*-band only, at λ = 330 nm (Figure 3b). The absorption at this point is dominated by glass substrate and MOF absorption a small part can be attributed to the AB. For each series of measurements, the data were fitted with Equation S3 and turned out, therefore, to be consistent with Malus' law.[10, 11]

$$A = A_0 + C\cos^2(\varphi_M - \varphi_0)/180°) \qquad \text{(S3)}$$

The fit parameters and their errors are given in Table S1. Equation S3 contains a constant term A_0 whose value depends strongly on wavelength of the last irradiation. The difference between UV and Vis measurements can therefore be used to describe the strength of optical changes in the system due to photoisomerization. The second term of the equation contains the $\cos^2$-dependence on φ_M due to photoalignment with the amplitude C and the phase φ_0. Deviations of the phase from 90° were likely caused by misaligned irradiation polarization and, possibly, by a small effect of the measurement light. The statistical fit errors are small in all cases.

Table S1. Parameters of fits in Figure 6 of the main work.

Type (plot legend)	A_0 / 10^{-3}	ΔA_0 / 10^{-3}	φ_0	$\Delta\varphi_0$	C / 10^{-3}	ΔC / 10^{-3}
LPUV (φ_{Irr} = 90°)	545	0.173	85	1.3	6.31	0.282
Vis	568	0.054	85	1.1	2.32	0.088
UV	546	0.177	71	7.6	1.09	0.290
Vis	568	0.105	78	3.1	1.58	0.172
LPUV (φ_{Irr} = 0°)	549	0.178	89	2.3	3.56	0.291
LPUV (φ_{Irr} = 90°)	544	0.104	84	0.9	5.58	0.170

3 References

(1) Yakovenko, A. A.; Reibenspies, J. H.; Bhuvanesh, N.; Zhou, H.-C., Generation and Applications of Structure Envelopes for Porous Metal–Organic Frameworks. *J. Appl. Crystallogr.* **2013,** *46*, 346-353.

(2) Müller, K.; Wadhwa, J.; Singh Malhi, J.; Schöttner, L.; Welle, A.; Schwartz, H.; Hermann, D.; Ruschewitz, U.; Heinke, L., Photoswitchable Nanoporous Films by Loading Azobenzene in Metal–Organic Frameworks of Type HKUST-1. *Chem. Commun.* **2017,** *53*, 8070-8073.

(3) Fu, W.-Q.; Liu, M.; Gu, Z.-G.; Chen, S.-M.; Zhang, J., Liquid Phase Epitaxial Growth and Optical Properties of Photochromic Guest-Encapsulated MOF Thin Film. *Cryst. Growth Des.* **2016,** *16*, 5487-5492.

(4) Konjhodzic, D.; Bretinger, H.; Marlow, F., Structure and Properties of Low-n Mesoporous Silica Films for Optical Applications. *Thin Solid Films* **2006,** *495*, 333-337.

(5) Rakhshani, A. E.; Makdisi, Y.; Ramazaniyan, H. A., Electronic and Optical Properties of Fluorine-Doped Tin Oxide Films. *J. Appl. Phys* **1998,** *83*, 1049-1057.

(6) Ching-Prado, E.; Watson, A.; Miranda, H., Optical and Electrical Properties of Fluorine Doped Tin Oxide Thin Film. *J. Mater. Sci. Mater. Electron.* **2018,** *29*, 15299-15306.

(7) Rau, H., Photoisomerization of Azobenzenes. In *Photochemistry and Photophysics*, Rabek, J. D., Ed. CRC Press: 1989.

(8) Knoll, H., Photoisomerism of Azobenzenes. In *CRC Handbook of Organic Photochemistry and Photobiology*, Horspool, W.; Lenci, F., Eds. CRC Press: 2004.

(9) Prestipino, C.; Regli, L.; Vitillo, J. G.; Bonino, F.; Damin, A.; Lamberti, C.; Zecchina, A.; Solari, P. L.; Kongshaug, K. O.; Bordiga, S., Local Structure of Framework Cu(II) in HKUST-1 Metallorganic Framework: Spectroscopic Characterization upon Activation and Interaction with Adsorbates. *Chem. Mater.* **2006,** *18*, 1337-1346.

(10) Eichler, H.-J.; Gobrecht, H.; Krystek, M.; Niedrig, H.; Richter, M.; Schoenebeck, H.; Weber, H.; Weber, K., *Bergmann Schäfer Lehrbuch der Experimentalphysik*. H. Gobrecht: New York, 1987; Vol. 8.

(11) Sekkat, Z.; Wood, J.; Knoll, W., Reorientation Mechanism of Azobenzenes within the Trans -> Cis Photoisomerization. *J. Phys. Chem.* **1995,** *99*, 17226-1723.
